# Supplementary figures and images for: Behavioral screening reveals a conserved residue in Y-Box RNA-binding protein required for associative learning and memory in C. elegans
Source: PLoS Genet. 2024 Oct 18;20(10):e1011443. doi: 10.1371/journal.pgen.1011443 (PMC11524487; doi:10.1371/journal.pgen.1011443)

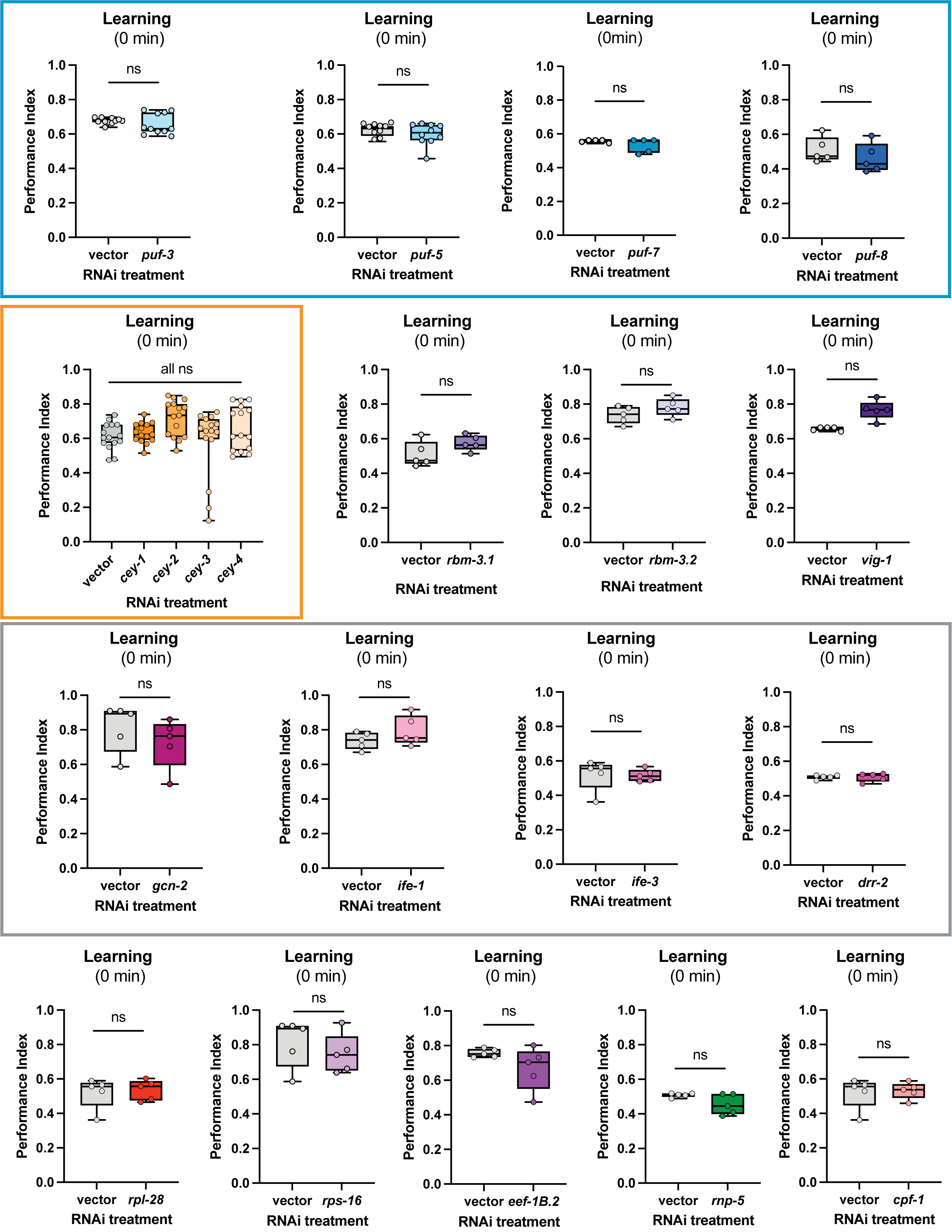

Supplement: S1 Fig — Boxes signify 3+ RBPs in the same protein family or class (blue box include PUF RBPs, orange box includes CEY RBPs, grey box includes translation initiation machinery). Box and whisker plots are shown for the learning timepoints from the STM/ITM assays for each RBP screened. Box and whisker plot: the center line denotes the median value (50th percentile) while the box contains the 25th to 75th percentiles. Whiskers mark the 5th and 95th percentiles. ns, not significant (p>0.05). (TIF) [file pgen.1011443.s001.tif]

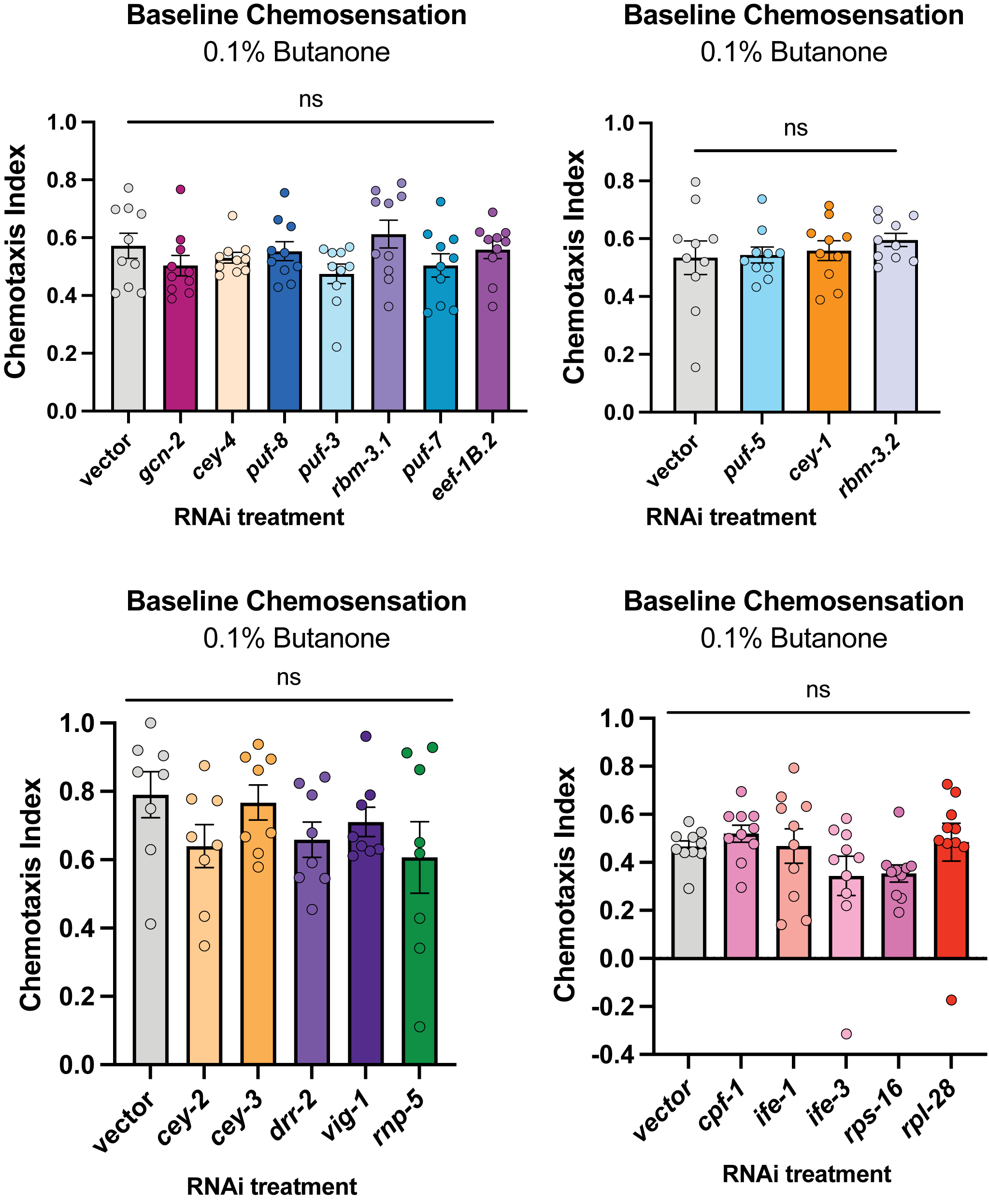

Supplement: S2 Fig — All RBPs in the screen are shown grouped by experiment. Bar represents mean. Whiskers mark the standard error of the mean. n = 10 per RNAi treatment. ns, not significant. (TIF) [file pgen.1011443.s002.tif]

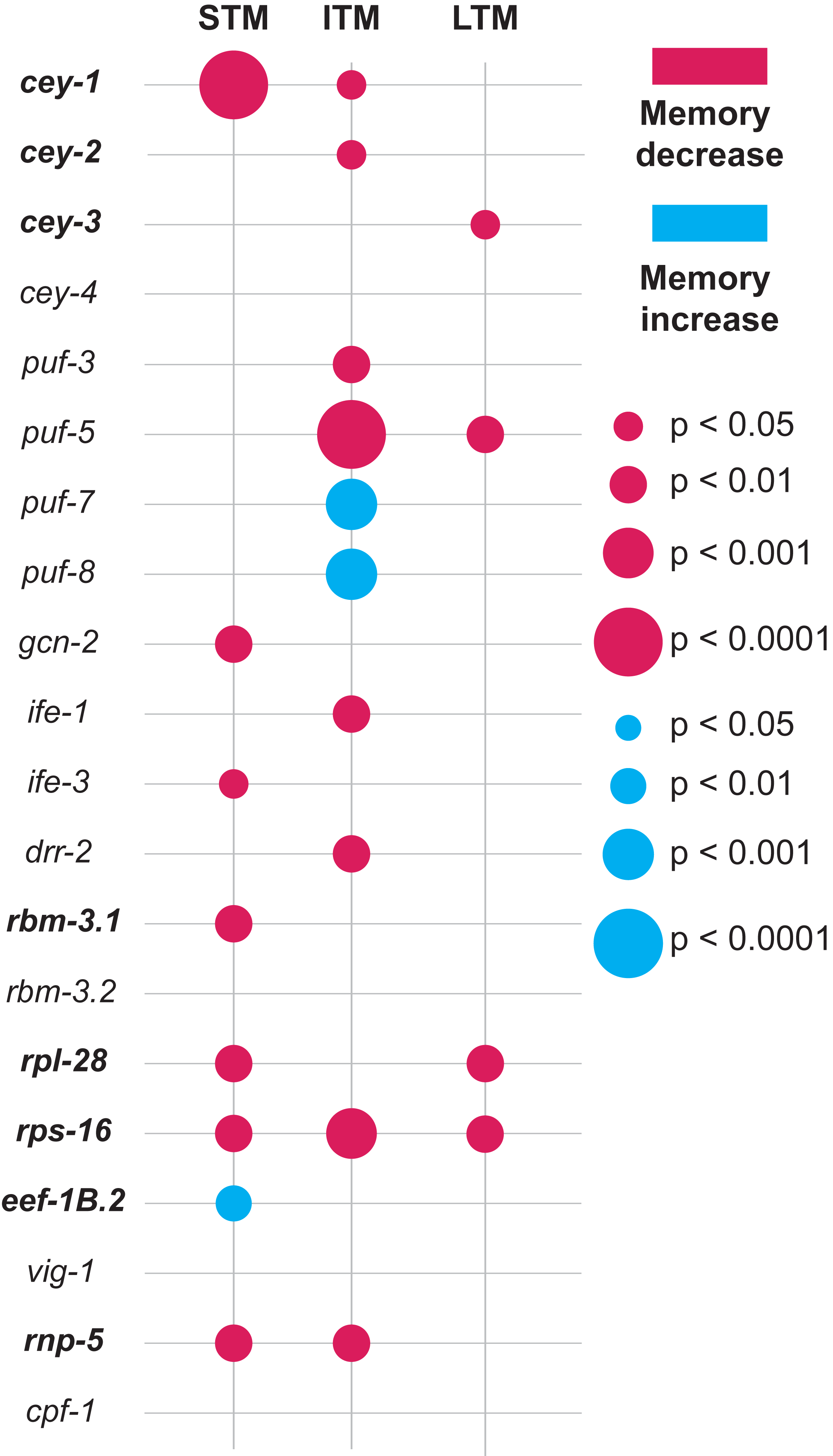

Supplement: S3 Fig — All RBPs are shown where pink circles are decreased memory and blue circles are increased memory. The size of circle represents the p value from a combined n ≥ 5–10 per RNAi treatment. (TIF) [file pgen.1011443.s003.tif]

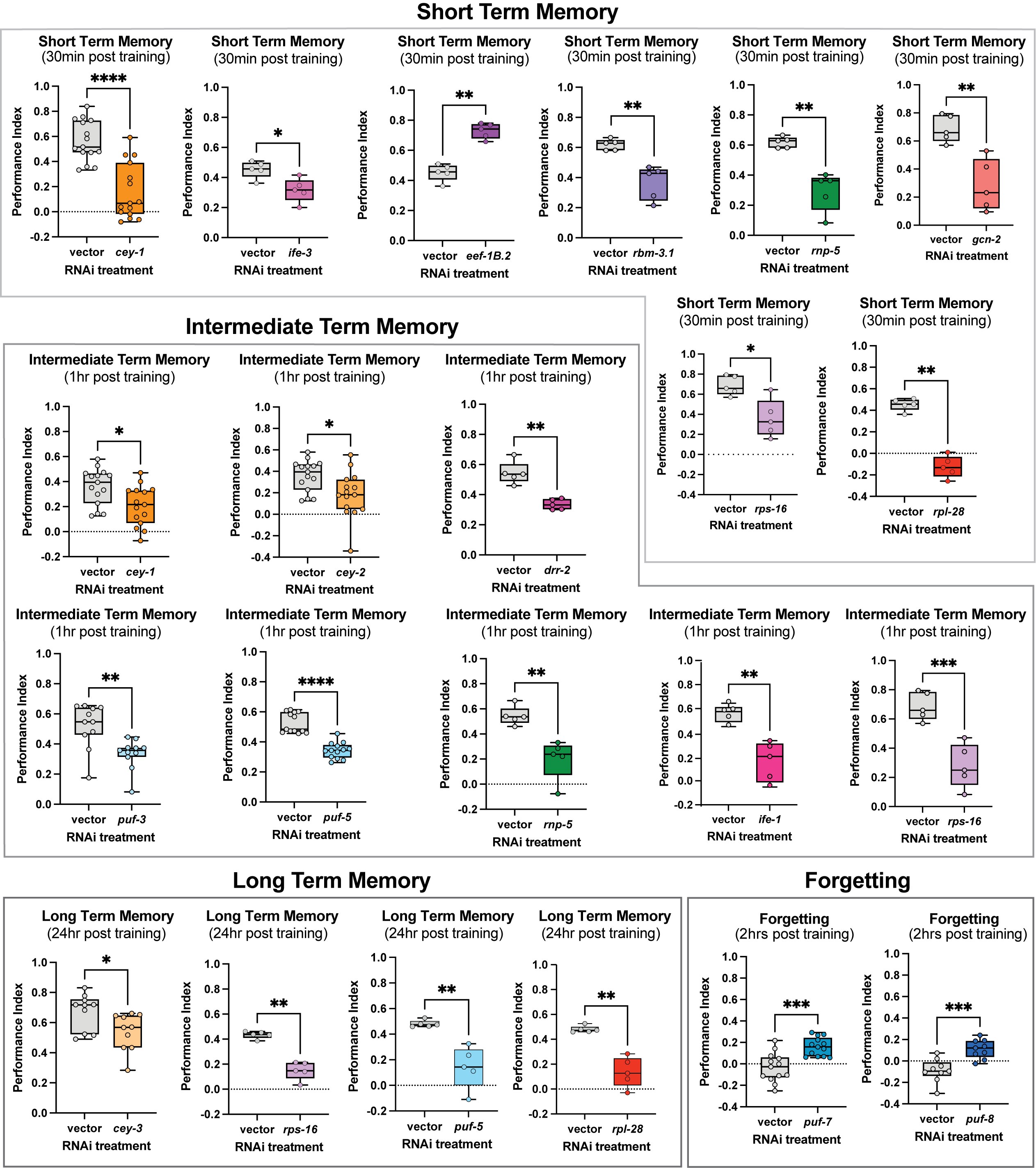

Supplement: S4 Fig — All RBPs with significantly altered memory are shown divided by memory timepoint. Box and whisker plot: the center line denotes the median value (50th percentile) while the box contains the 25th to 75th percentiles. Whiskers mark the 5th and 95th percentiles. n ≥ 5–10 per RNAi treatment. *p<0.05,**p<0.01, ***p<0.001, ****p<0.0001. (TIF) [file pgen.1011443.s004.tif]

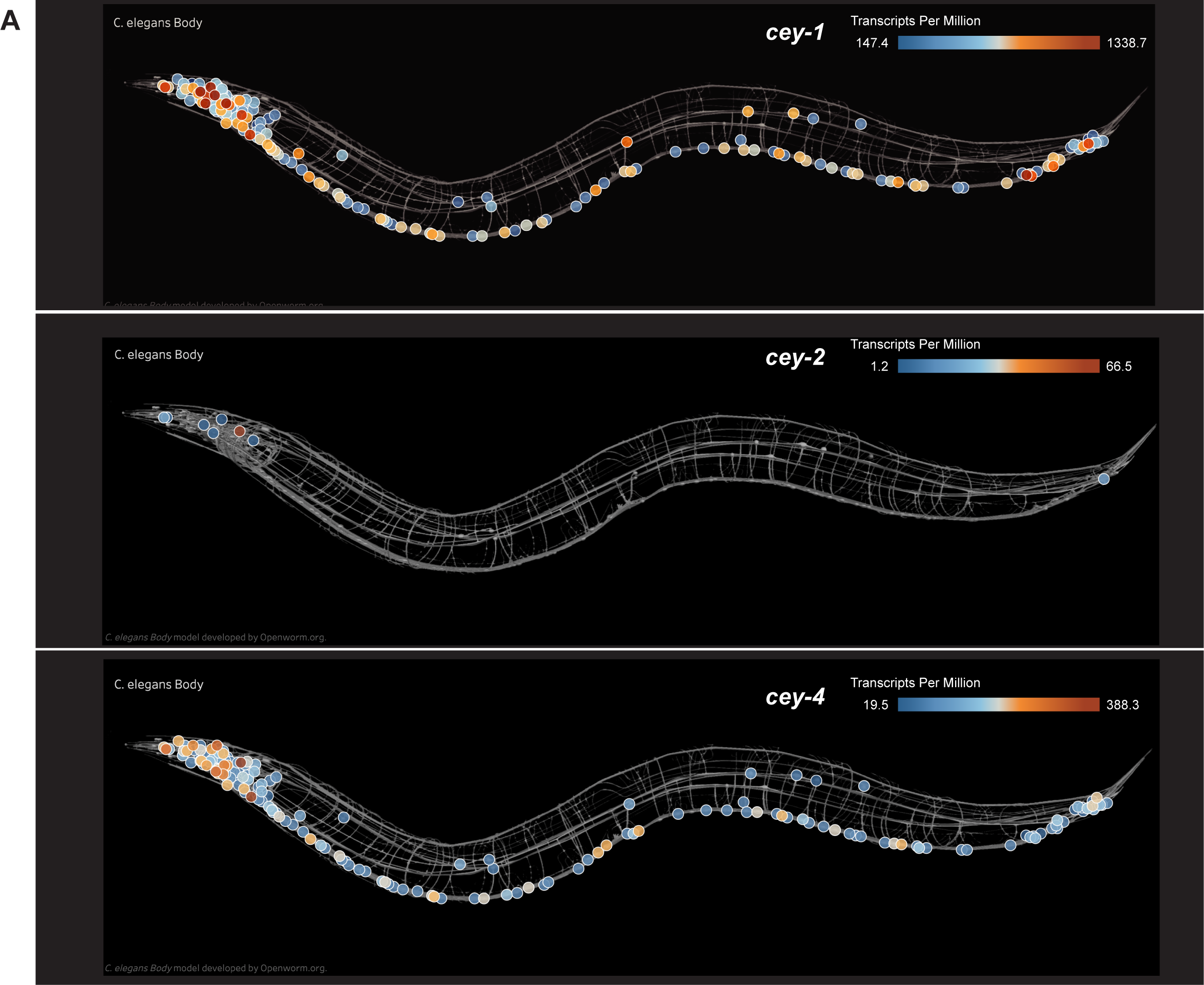

Supplement: S5 Fig — While cey-1 and cey-4 have broad expression, cey-2 is only located in eight neurons at L4. (TIF) [file pgen.1011443.s005.tif]

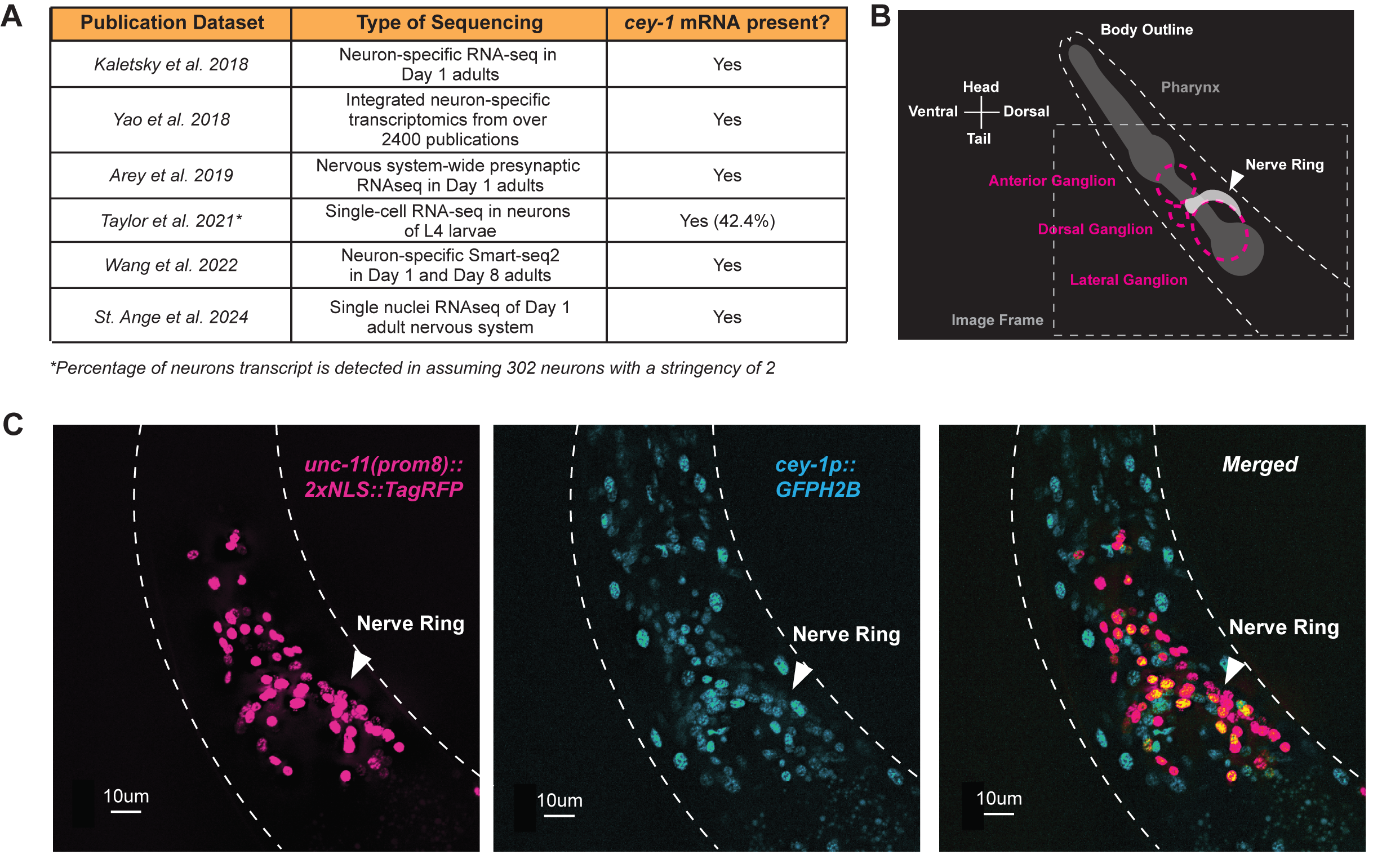

Supplement: S6 Fig — (A) Neuron-specific and/or single-cell RNA-seq data compiled from five different publications suggest cey-1 mRNA is expressed in the adult nervous system [24, 35, 38, 43, 80, 137, 138]. (B) Diagram of microscopy images shown in (C). C. elegans head is labeled including the location of the pharynx and main neuronal ganglia/nerve rings. (C) A transcriptional cey-1 reporter suggests the gene is broadly expressed in the neurons in the head at baseline conditions. Representative image of Day 2 adult worms with RFP-labeled neuronal nuclei (unc-11(prom8)::2xNLS::TagRFP)) pseudocolored magenta and a nuclear GFPH2B cey-1 promoter fusion (cey1p::GFPH2B) pseudocolored cyan show colocalization. (TIF) [file pgen.1011443.s006.tif]

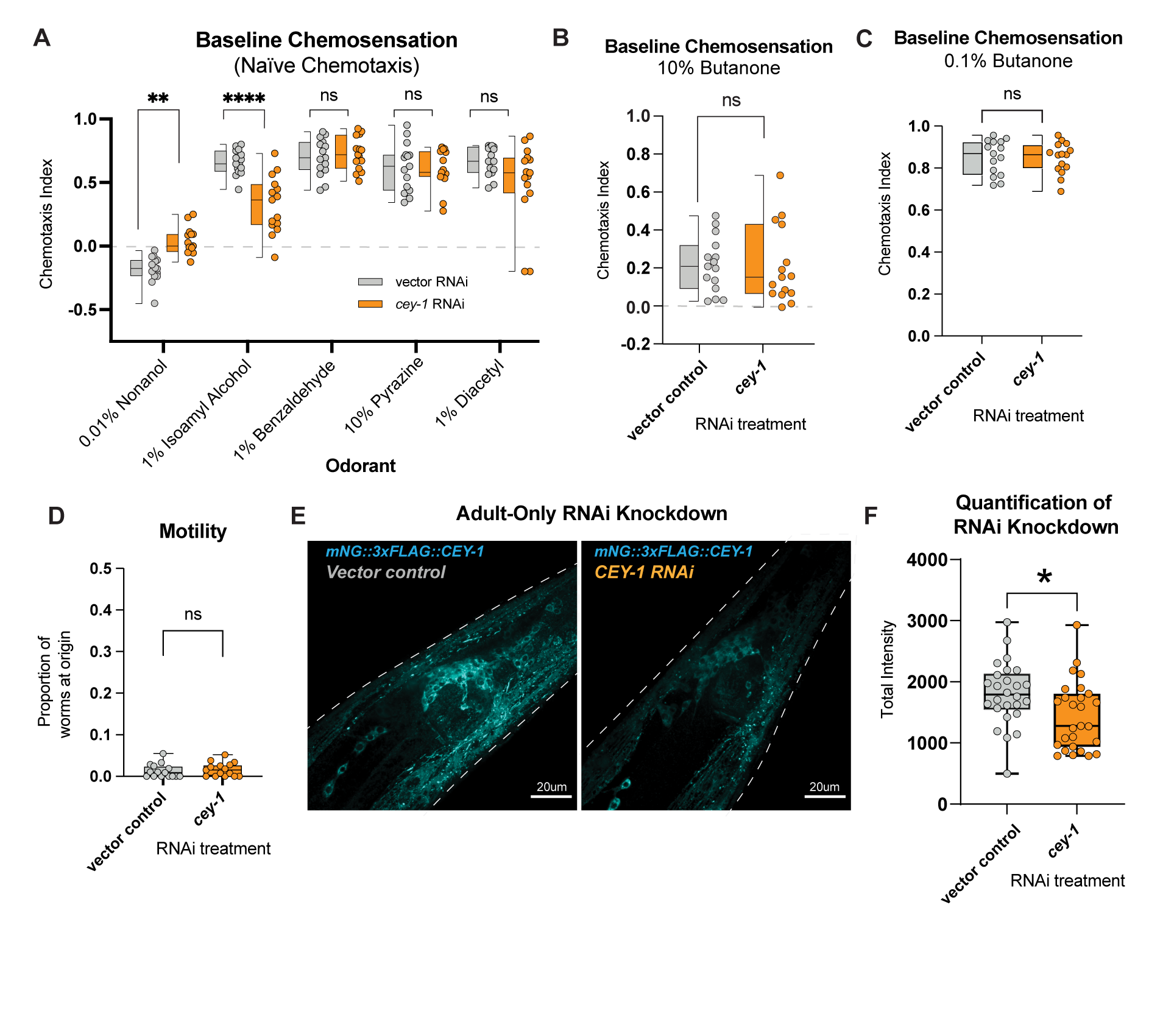

Supplement: S7 Fig — (A) Naïve battery of both negative and positive odors reveals deficits in both nonanol and isoamyl alcohol chemotaxis, but not in benzaldehyde, pyrazine, or diacetyl chemotaxis at attractive concentrations. (B) Baseline chemosensation for butanone at neutral concentration of 10% is unaffected by neuron-specific knockdown of cey-1. n = 15 per RNAi treatment. (C) Baseline chemosensation for butanone at an attractive concentration of 0.1% is unaffected by neuron-specific knockdown of cey-1. n = 15 per RNAi treatment. (D) Motility, measured as proportion of worms at the origin of a chemotaxis plate, is unaffected by neuron-specific loss of cey-1. n = 15 per RNAi treatment. Box and whisker plot: the center line denotes the median value (50th percentile) while the box contains the 25th to 75th percentiles. Whiskers mark the 5th and 95th percentiles. **p<0.01, ****p<0.0001. ns, not significant (p>0.05). (E) Representative images of adult-only, neuron-specific knockdown RNAi treatment (vector or cey-1 RNAi) in neuronally RNAi-sensitized mNG::CEY-1 animals. (F) Quantification of total fluorescent intensity. n = 26 for each RNAi condition. *p<0.05. (TIF) [file pgen.1011443.s007.tif]

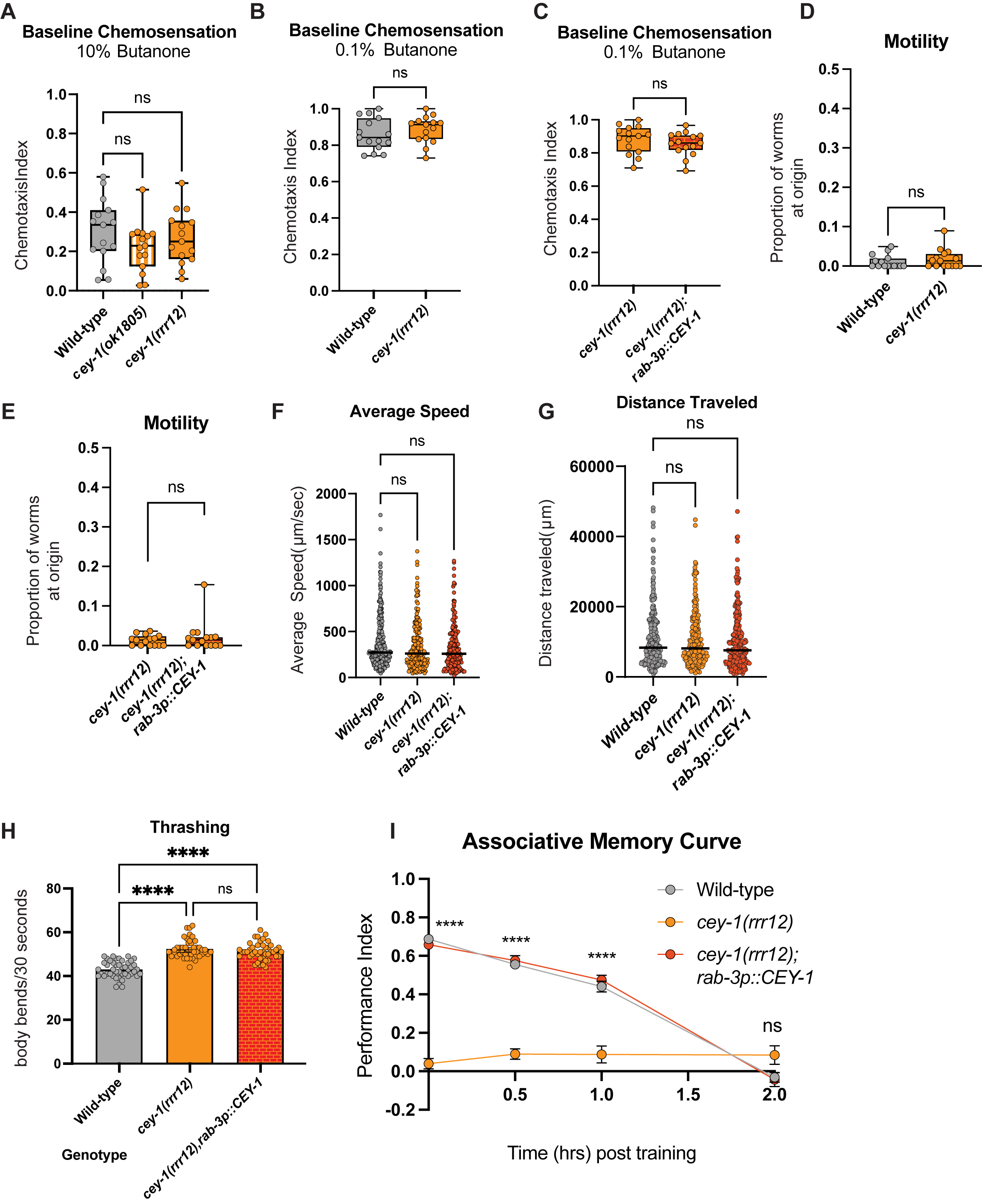

Supplement: S8 Fig — (A) Naïve chemotaxis towards 10% butanone prior to behavioral conditioning does not significantly differ between wild-type animals, cey-1(ok1805), and cey-1(rrr12) mutants. n = 15 per genotype. (B) Baseline chemosensation for butanone at an attractive concentration of 0.1% is unaffected by whole-body loss of cey-1. n = 15 per genotype. (C) Baseline chemosensation for butanone at an attractive concentration of 0.1% is unaffected by neuron-specific rescue of cey-1. n = 15 per genotype. (D) Motility, measured as proportion of worms at the origin of a chemotaxis plate, is unaffected by whole-body loss of cey-1. n = 15 per genotype. (E) Motility, measured as proportion of worms at the origin of a chemotaxis plate, is unaffected by neuron-specific rescue of cey-1. n = 15 per genotype. (F) Day 2 adult speed measured in μm/sec by Wrmtrck. n = 20–30 worms per genotype, Tracks were thresholded at 15 seconds duration, with multiple tracks per worm possible. ns, not significant. (G) Day 2 average track distance (in μm) traveled as measured by Wrmtrck. n = 20–30 worms per genotype, Tracks were thresholded at 15 seconds duration, with multiple tracks per worm possible. ns, not significant. Of note, for both F and G, wild-type controls for this figure are the same as S11 Fig because experiments were performed the same day each time; data was put into two separate graphs for organization. (H) Day 2 thrashing comparing number of body bends per 30 seconds of wild-type, cey-1(rrr12) knockout worms, and nervous system specific rescue worms (cey-1(rrr12);rab-3p::CEY-1) shows that cey-1 mutants do not have thrashing deficits, and indeed exhibit more body bends per minute. n = 50 for each genotype. (I) Associative memory curve comparing wild-type, cey-1(rrr12) knockout worms, and nervous system specific rescue worms (cey-1(rrr12);rab-3p::CEY-1) shows that while knockouts have no associative learning or memory, neuron-specific rescue of cey-1 allows for learning and memory equivalent to wi [file pgen.1011443.s008.tif]

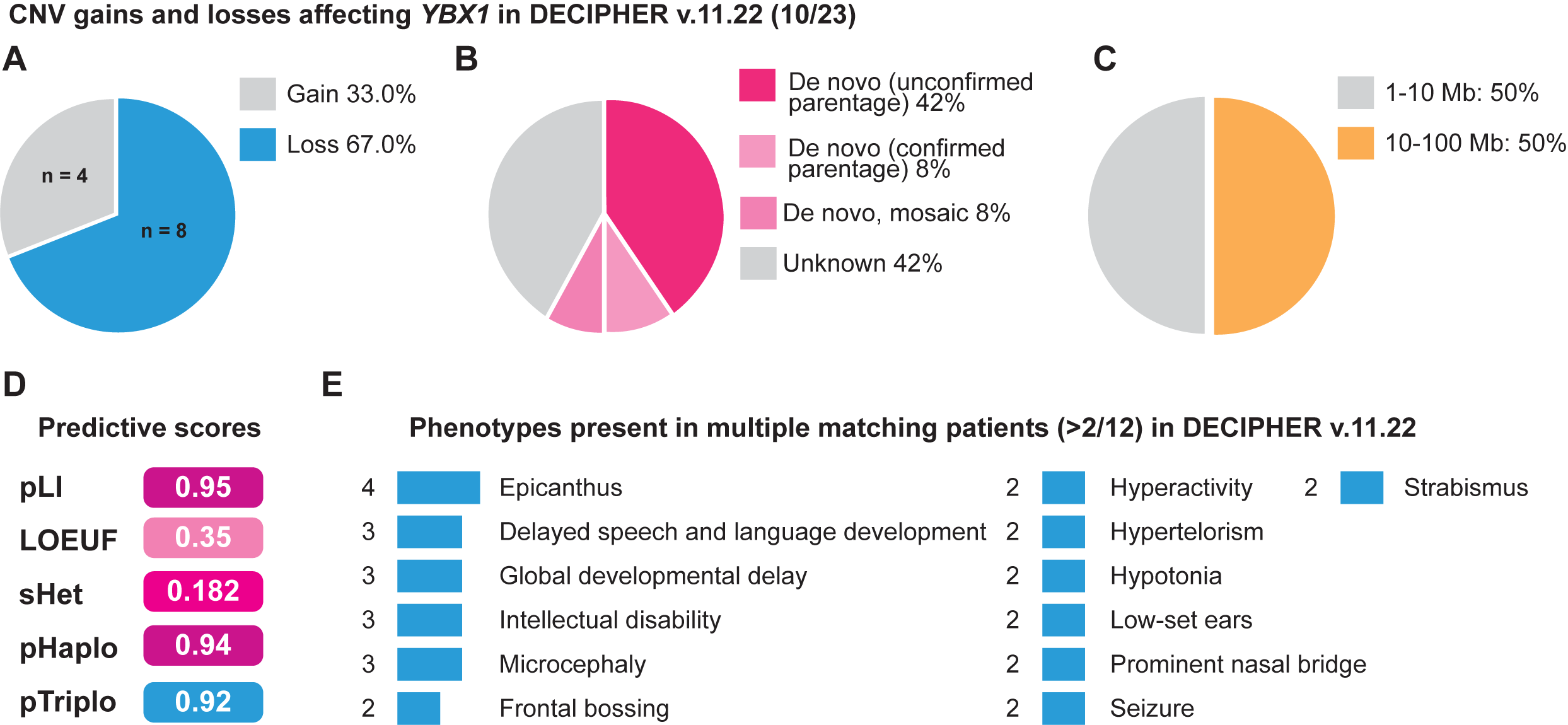

Supplement: S9 Fig — (A) Percentages of individuals reported with gain or loss CNVs that include YBX1. (B) Percentages of mechanisms of inheritance of gain and loss CNVs that include YBX1. (C) Size of CNV gain and losses that include YBX1. (D) Predictive scores for YBX1 from gnomAD v.2.11 [132] suggest that YBX1 is intolerant to loss of function variants and is haploinsufficient. (E) Phenotypes of patients specifically with deletion/loss CNVs including YBX1 include epicanthus, delayed speech and development, intellectual disability, and other neurological features. (TIF) [file pgen.1011443.s009.tif]

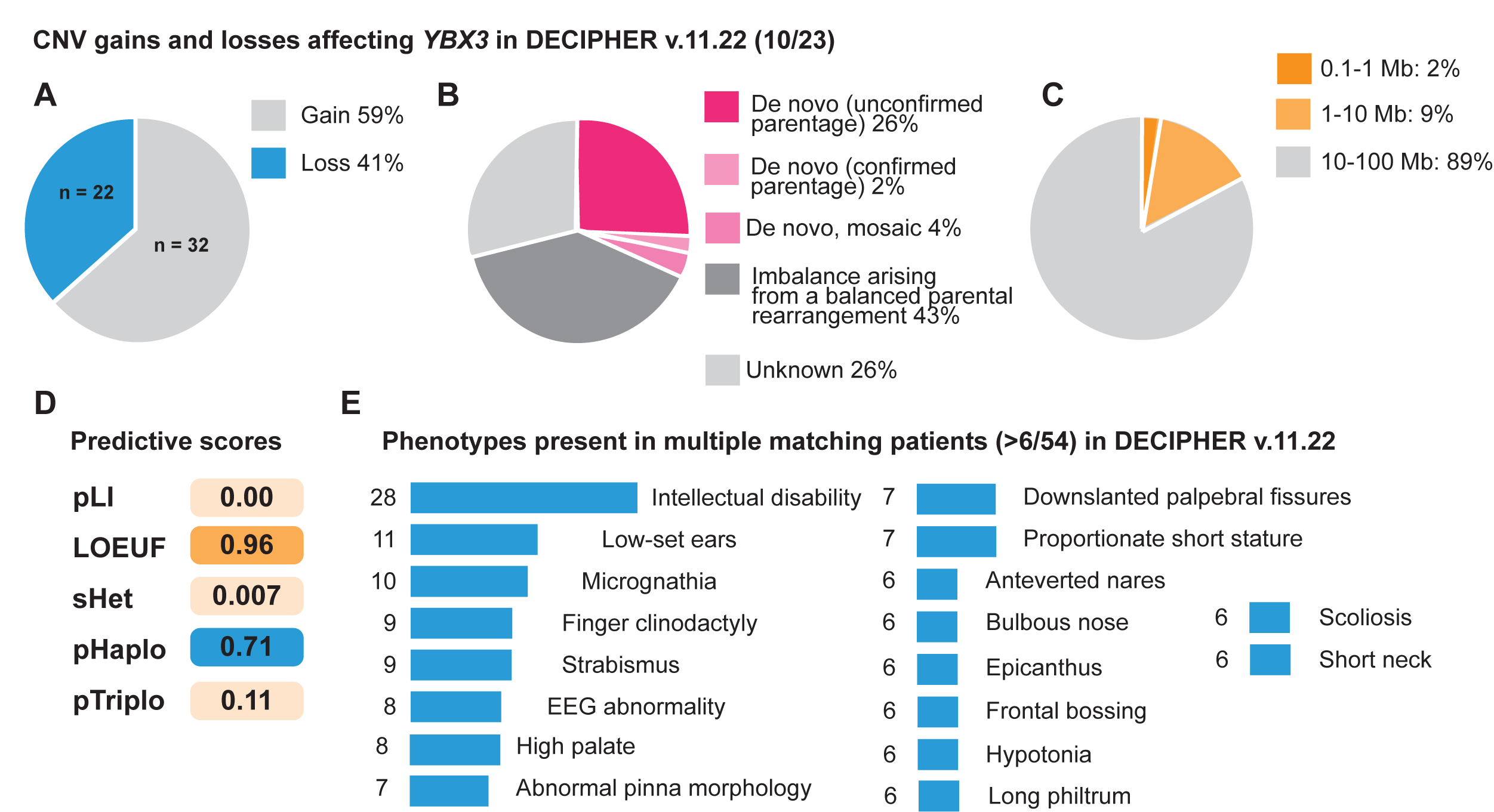

Supplement: S10 Fig — (A) Percentages of individuals reported with gain or loss CNVs that include YBX3. (B) Percentages of mechanisms of inheritance of gain and loss CNVs that include YBX3. (C) Size of CNV gain and losses that include YBX3. (D) Predictive scores for YBX3 from gnomAD v.2.11 [132] suggest that YBX3 is tolerant to loss of function variants and is haplosufficient, suggesting variants may instead be deleterious by being dominant negative or gain-of-function. (E) Phenotypes of patients specifically with deletion/loss CNVs including YBX3, primarily intellectual disability, low-set ears, micrognathia, and other neurological features. (TIF) [file pgen.1011443.s010.tif]

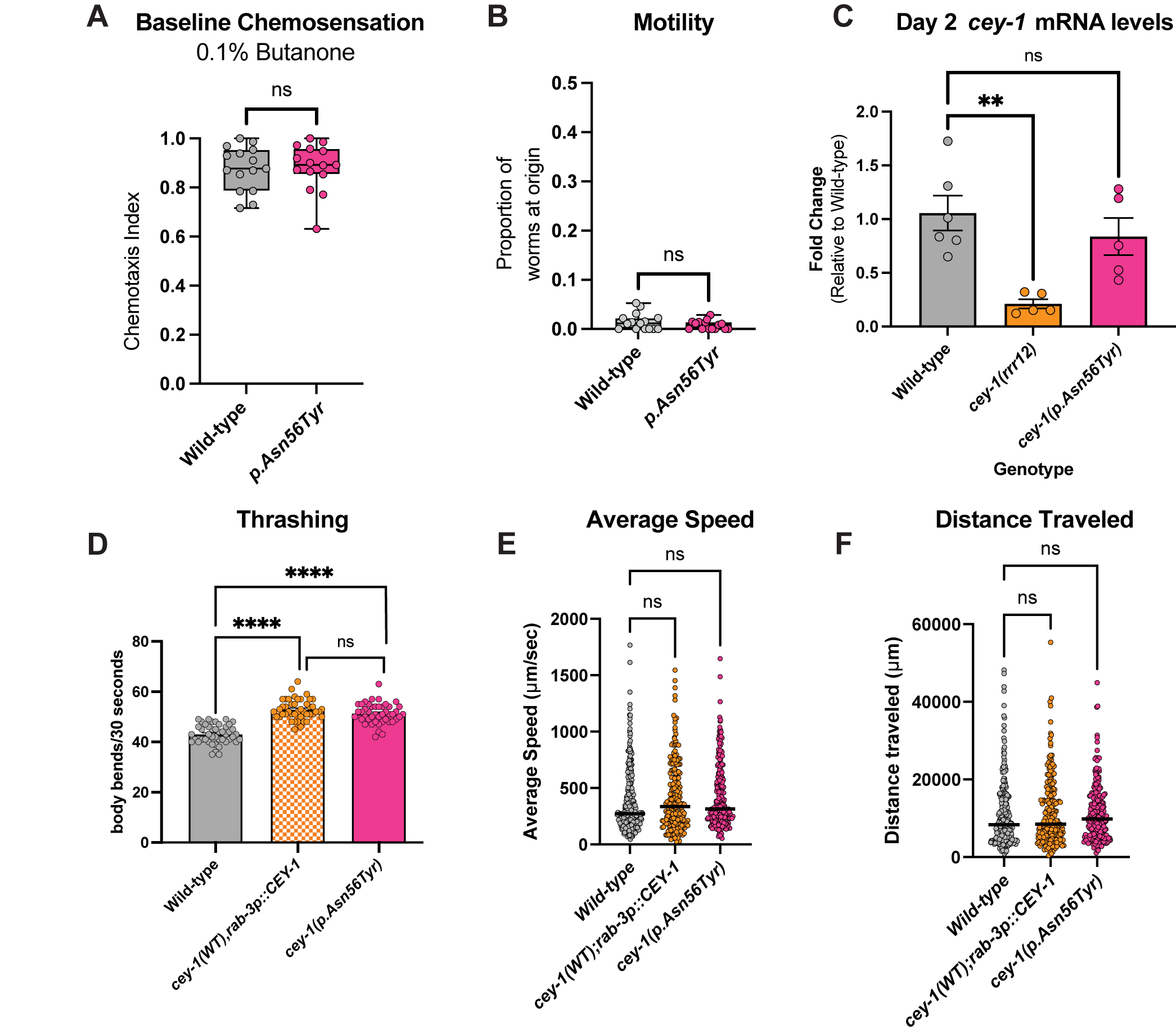

Supplement: S11 Fig — (A) Baseline chemosensation for butanone at an attractive concentration of 0. 1% is unaffected by the p.Asn56Tyr variant in cey-1. n = 15 per genotype. (B) Motility, measured as proportion of worms at the origin of a chemotaxis plate, is unaffected by p.Asn56Tyr variant in cey-1. n = 15 per genotype. Box and whisker plot: the center line denotes the median value (50th percentile) while the box contains the 25th to 75th percentiles. Whiskers mark the 5th and 95th percentiles. ns, not significant (p>0.05). (C) qRT-PCR of cey-1 mRNA levels in Day 2 adults shows that while cey-1 knockouts (cey-1(rrr12)) worms undergo nonsense mediated decay [56] resulting in reduced cey-1 mRNA levels, introduction of the p.Asn56Tyr variant has no significant effect on cey-1 expression. n = 6 per genotype. **p<0.01. (D) Day 2 thrashing comparing number of body bends per 30 seconds of wild-type, neuron-specific CEY-1 overexpression (cey-1(WT);rab-3p::CEY-1), and variant worms with p.Asn56Tyr in endogenous CEY-1 (cey-1(p.Asn127Tyr) shows that cey-1 mutants do not have thrashing deficits, and indeed exhibit more body bends per minute. n = 50 for each genotype. Of note, wild-type controls for this figure are the same as S8 Fig because experiments were performed the same day each time; data was put into two separate graphs for organization. (E) Day 2 adult speed measured in μm/sec by Wrmtrck. n = 20–30 worms per genotype, Tracks were thresholded at 15 seconds duration, with multiple tracks per worm possible. ns, not significant. (F) Day 2 average track distance (in μm) traveled as measured by Wrmtrck. n = 20–30 worms per genotype, Tracks were thresholded at 15 seconds duration, with multiple tracks per worm possible. ns, not significant. Of note, for both E and F, wild-type controls for this figure are the same as S8 Fig because experiments were performed the same day each time; data was put into two separate graphs for organization. (TIF) [file pgen.1011443.s011.tif]

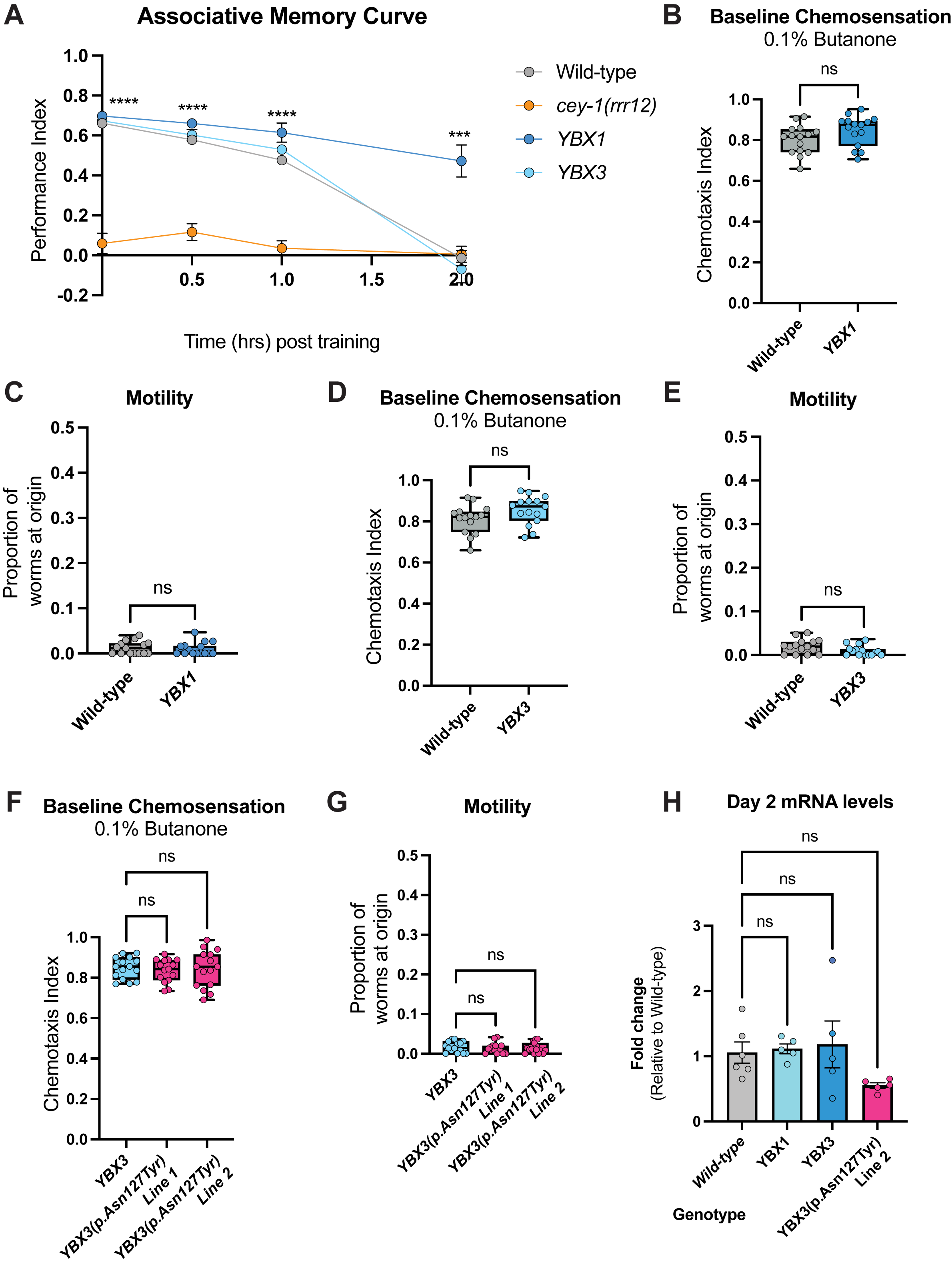

Supplement: S12 Fig — (A) Associative memory curve comparing wild-type, cey-1 knockouts, and humanized lines expressing either YBX1 or YBX3 at the endogenous cey-1 locus. While worms expressing YBX3 have normal memory performance, those expressing YBX1 appear to have extended memory, as they have a strong association for butanone even two hours post-training. (B) Baseline chemosensation for butanone at an attractive concentration of 0.1% is normal in worms expressing YBX1. n = 15 per genotype. (C) Motility, measured as proportion of worms at the origin of a chemotaxis plate, is unaffected by YBX1. n = 15 per genotype. (D) Baseline chemosensation is normal in worms expressing YBX3 at the cey-1 locus. n = 15 per genotype. (E) Motility is unaffected by YBX3. n = 15 per genotype. (F) Baseline chemosensation is unaffected by expressing YBX3(p.Asn127Tyr) at the cey-1 locus. n = 15 per genotype. (G) Motility is unaffected by YBX3(p.Asn127Tyr). n = 15 per genotype. (H) qRT-PCR of cey-1 and YBX mRNA levels in Day 2 adults shows that levels of YBX1, YBX3, and YBX3(pAsn127Tyr) are not significantly reduced compared to wild-type cey-1 mRNA levels and therefore, introduction of the p.Asn127Tyr variant has no significant effect on expression. n = 5 per genotype. ns, not significant. (TIF) [file pgen.1011443.s012.tif]

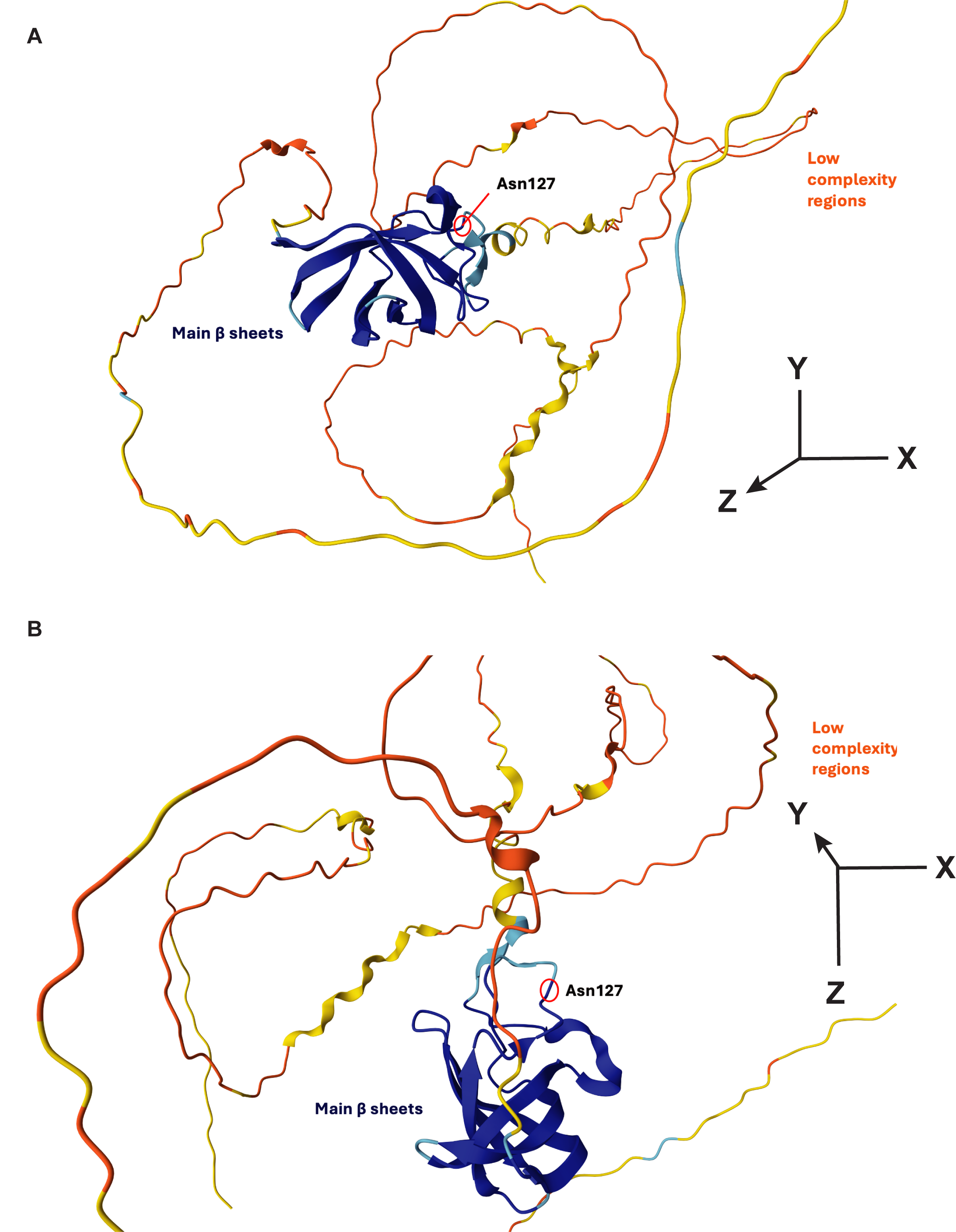

Supplement: S13 Fig — (A) Forward-facing view of the protein with the β sheet that binds RNA facing the viewer. (B) Top-down view of the YBX3 protein depicts the location of the Asn127 residue. (TIF) [file pgen.1011443.s013.tif]
